# Gastroprotective Mechanism and Ulcer Resolution Effect of* Cyrtocarpa procera* Methanolic Extract on Ethanol-Induced Gastric Injury

**DOI:** 10.1155/2018/2862706

**Published:** 2018-01-09

**Authors:** Wendy Itzel Escobedo-Hinojosa, Erika Gomez-Chang, Karina García-Martínez, Raquel Guerrero Alquicira, Alexandre Cardoso-Taketa, Irma Romero

**Affiliations:** ^1^Departamento de Bioquímica, Facultad de Medicina, Universidad Nacional Autónoma de México, Ciudad Universitaria, 04510 Ciudad de México, Mexico; ^2^Departamento de Biología Celular y Tisular, Facultad de Medicina, Universidad Nacional Autónoma de México, Ciudad Universitaria, 04510 Ciudad de México, Mexico; ^3^Centro de Investigación en Biotecnología, Universidad Autónoma del Estado de Morelos, Av. Universidad 1001, Col. Chamilpa, 62209 Cuernavaca, MOR, Mexico

## Abstract

Gastric ulcers are a worldwide health problem and their poor healing is one of the most important causes for their recurrence. We have previously reported the remarkable gastroprotective and anti-*Helicobacter pylori* activities of the methanolic extract (CpMet) of* Cyrtocarpa procera* bark. This work investigates, in a murine model, the CpMet gastroprotective mechanism and establishes its preclinical efficacy in the resolution of ethanol-induced gastric ulcers. The results showed that the gastroprotective activity of CpMet is mainly associated with endogenous NO and prostaglandins, followed by sulfhydryl groups and K_ATP_ channels. Furthermore, CpMet (300 mg/kg, twice a day) orally administered during 20 consecutive days promoted an ulcer area reduction of 62.65% at the 20th day of the treatment. The effect was confirmed macroscopically by the alleviation of gastric mucosal erosions and microscopically by an increase in mucin content and a reduction in the inflammatory infiltration at the site of the ulcer. No clinical symptoms or signs of toxicity were observed in the treated animals. The results indicate the safety and efficacy of CpMet in promoting high quality of ulcer healing by different mechanisms, but mostly through cytoprotective and anti-inflammatory effects, making it a promising phytodrug for ulcer treatment.

## 1. Introduction

Gastric ulcers (GU) are open sores in the lining of the stomach that extend to or beyond the muscularis mucosa. The incidence of GU varies widely around the world depending on the age, gender, and geographical location but they remain a very common condition worldwide and a major public health problem due to high healthcare costs and mostly to life-threatening complications such as bleeding, perforation, and obstruction, which explains the high morbidity and mortality associated with this disease [1–4].

The pathophysiology of gastric ulceration is multifactorial but is generally considered as a result of an imbalance in the equilibrium between protective and aggressive factors of the gastric mucosa [5]. The gastrointestinal defense mechanisms include gastric mucosal integrity, mucus secretion, bicarbonate production, nitric oxide (NO), gastroprotective prostaglandin synthesis, normal gastric motility, and adequate tissue microcirculation, while the noxious factors comprise, among others, gastric acid and pepsin secretion, bile salts, reactive oxygen species (ROS),* Helicobacter pylori* infection, alcohol consumption, and prolonged ingestion of nonsteroidal anti-inflammatory (NSAIDs) drugs [6–8].

As expected, the current treatments of GU are targeted for either enhancing gastric mucosal defenses or counteracting injurious factors. The hallmark drugs have been the ones that reduce gastric acid secretion such as H_2_-receptor antagonists (e.g., ranitidine) and proton pump inhibitors (e.g., omeprazole) as well as antibiotic therapy for* H. pylori* eradication [3]. Despite the fact that acid antisecretory drugs have been a cornerstone in the treatment of this pathology, the high costs and side effects of long-term regimens combined with ulcer recurrence and some cases of refractoriness to conventional acid suppression therapies urge to search for new antiulcer agents addressed to enhance the healing of GU with fewer disadvantages than current treatments [9, 10]. The quality of ulcer healing (QOUH) is a key point in the pathogenesis of gastric ulcers since it has been reported that abnormalities in mucosal regeneration within the scars of healed ulcers, as well as the persistence of chronic inflammation demonstrated by the presence of increased infiltration of neutrophils and macrophages, are the basis for ulcer recurrence [11]. Therefore, the research of new therapeutic agents should also focus on improving the QOUH. In this sense, herbal drugs have become excellent sources for the development of new treatments to heal GU since they are effective, reduce the offensive factors, appear to be safer, have better tolerance in patients, and are less expensive for the populations [12, 13].

*Cyrtocarpa procera* Kunth (Anacardiaceae) commonly known as “chupandilla,” “copalcojote,” or “coco de cerro” is endemic to Mexico. It is a tree mainly distributed in the deciduous and subdeciduous dry forests of the states of Jalisco, Michoacán, Nayarit, Guerrero, Oaxaca, México, Morelos, and Puebla. The aqueous decoction and infusion of* C. procera* bark have been extensively used in Mexican folk medicine for digestive disorders such as dysentery and diarrhea, for kidney ailments, and for toothaches, among other uses [14–16]. Due to its similar appearance,* C. procera* bark is used to adulterate the* Amphipterygium adstringens* bark, one of the most important and commercialized Mexican medicinal plants used to treat gastritis, gastric ulcer, and stomach cancer [14, 17].

Only few studies have addressed the phytochemical and pharmacological properties of* C. procera* bark. Phytochemical studies have reported the isolation and identification of some sterols such as *β*-amyrin and *β*-sitosterol, and the fatty acids 1,3-propyl-dipentadecanoate, 3-hydroxypropyl-9-octadecenoate, pentadecylbenzene, eicosylbenzene, docosane, heptacosane, dotriacontane, and 2,6,10-trimethyltetradecane [18, 19]. Concerning the pharmacological information, it has been explored the antiulcerogenic activity of* C. procera* bark extracts in ethanol-induced gastric ulcers in a rat model [19] and the antibiotic activity of a methanolic extract against a range of Gram-positive and Gram-negative bacteria [15]. In the latter case, our group has reported the anti-*Helicobacter pylori* activity of aqueous and methanolic extracts of* C. procera* [20].

In our previous work with this plant [21] we made a systematic evaluation of the anti-*H. pylori*, anti-inflammatory and gastroprotective properties of different polarity* C. procera* bark extracts in order to identify if any of them had polypharmalcological effects. The results demonstrated a remarkable gastroprotective activity of the methanolic extract (ED_50_ = 0.53 mg/kg) in an acute ethanol ulcer model and a good* in vitro* anti-*H. pylori* activity (MIC = 62.5 *μ*g/ml) being more effective than the reference antibiotic metronidazole. Also, the methanolic extract was not toxic under acute administration and had high yield, making it a good candidate for further clinical studies.

Thus, in order to further clarify the efficacy of the methanolic extract of* C. procera (CpMet) *as an antiulcerative agent, the present work was undertaken to investigate the gastroprotective mechanism and to establish its preclinical efficacy in the resolution of gastric ulcers induced by ethanol as well as its toxicological safety during a 20-day repeated-dose oral administration.

## 2. Materials and Methods

### 2.1. Plant Material

The collection of* C. procera* stem bark was made in Jojutla, Morelos, in the Higueron Locality (18°37′12.5^″^ N, 99°10′33.7^″^ W), on March 2010, and it was identified by Mtra. Rosa María Fonseca from the Faculty of Sciences, Universidad Nacional Autónoma de México (UNAM). The species vouchers specimens 125630 and 125636 were deposited in the Faculty of Sciences Herbarium, UNAM. The name of the plant has been checked as an accepted name on http://www.theplantlist.org (accessing date 26/08/17).

### 2.2. Preparation of Methanolic Extract

The methanolic extract* (CpMet)* was prepared by exhaustive maceration of 1.5 kg of dried milled vegetable material with methanol (1 : 10 w/v). The solvent was separated from the residue by filtration and then evaporated to dryness under reduced pressure. 431 g of* CpMet*, equivalent to 28.7% of dry weight yield, was obtained. The extract was kept at room temperature in the dark until it was used.

### 2.3. Animals

Male CD-1 mice from UNAM's Faculty of Medicine vivarium weighting 20–25 g were used for the toxicity evaluation and 35–40 g mice for the rest of the experiments. They were housed in standard laboratory conditions (22 ± 2°C, 12-h light dark cycle), with free access to standard pellet diet (Rodent Lab Chow Purina®) and water ad libitum. 12 hours prior to each experiment, the animals were deprived of food with free access to water and placed in individual wire-net raised floors to prevent coprophagy. The experimental protocols were approved by the Ethical Committee of the UNAM's Faculty of Medicine (approval number 087/2013) and conducted in conformity with the Mexican Official Norm for animal care and handling (NOM-062-ZOO-1999) and in accordance with the ethical guidelines of the National Institutes of Health. The assays recorded in this work required different number of animals as indicated below.

### 2.4. Chemicals and Drugs

The methanolic extract of* C. procera* was dissolved in 0.9% NaCl (isotonic saline solution). Absolute ethanol (ETOH), N^*ω*^-nitro-L-arginine methyl ester (L-NAME), N-ethylmaleimide (NEM), glibenclamide (GLIB), indomethacin (INDO), and carbenoxolone (CAR) were purchased from Sigma-Aldrich Co. Omeprazole (OME) was obtained from Liomont®. Ketamine and Xylazine were acquired from Pisa®.

All the samples administered by intragastric route, independently of the final concentration used, were always suspended in a volume of 10 ml/kg.

### 2.5. Ethanol-Induced Gastric Mucosal Lesions in N^*ω*^-Nitro-L-arginine Methyl Ester, Indomethacin, N-Ethylmaleimide, and Glibenclamide-Pretreated Mice

In order to investigate the* CpMet* gastroprotective mechanism of action, separate experiments were conducted using the following drugs to pretreat the animals: INDO, a prostaglandin synthesis inhibitor (10 mg/kg dissolved in 5 mM NaHCO_3_, s.c.); NEM, a sulfhydryl compound blocker (1 mg/kg dissolved in isotonic saline solution, s.c.); L-NAME, a nitric oxide synthase inhibitor (10 mg/kg dissolved in isotonic saline solution, i.p.); and GLIB, an ATP-sensitive potassium channel blocker (5 mg/kg dissolved in isotonic saline solution, i.g.). The corresponding vehicle, either 5 mM NaHCO_3_ or isotonic saline solution, was administered in control groups. The animal groups (*n* = 6) received the pretreatments 30 min before the intragastric administration of the respective treatment, either isotonic saline solution (10 ml/kg) or* CpMet *(100 mg/kg). An hour later, the ulceration was induced according to the method described by Bucciarelli and Skliar [22] by intragastric instillation of ETOH at a dose of 7 ml/kg of body weight. An hour and a half after ETOH administration, animals were sacrificed by carbon dioxide inhalation in an appropriate chamber. Each stomach was dissected out, insufflated with 2 ml of 10% formalin, and fixed in the same solution for 15 min. The stomachs were opened along the greater curvature, pressed between two glass plates, and scanned. The lesion area was determined with the aid of a public domain Java image processing program (Image J) developed at the US National Institutes of Health (freely available at https://rsb.info.nih.gov/nih-image/). The sum of the areas of all ulcers in the corpus of each stomach was considered as the lesion area (mm^2^). At least three independent experiments were performed.

### 2.6. pH Measurement of Gastric Contents

In order to determine whether the oral administration of* CpMet* modifies the pH value of gastric contents, the following assay was performed according to the method described by Shay et al. [23] with modifications. Briefly, after 12 h of fasting, the animals were separated into different groups (*n* = 6) and the treatments (*CpMet* 100 mg/kg; omeprazole 20 mg/kg) or the vehicle (10 ml/kg) was administrated by oral gavage. One hour later, mice were anesthetized with ketamine/xylazine (150/10 mg/kg, i.p.). The abdomen was opened through a midline epigastric incision, and the pyloric portion was occluded with a ligature. The wound was closed and the animals were placed back in their cages to recover from anesthesia. After 20 min, the mice were treated with VEH (isotonic saline solution, 7 ml/kg, i.g.) or with ETOH (7 ml/kg). Four hours later, mice were sacrificed, the abdomen opened, and the stomach was removed after clamping the pylorus and the lower end of the esophagus. By opening the stomach along its greater curvature the contents were collected in a graduated centrifuge tube. The gastric contents were centrifuged at 3,000 rpm (8,000 ×g, 25°C) for 10 min. The gastric pH values were measured with a pH meter (HANNA Instruments pH 211).

### 2.7. Repeated-Dose 20-Day Toxicity Evaluation of CpMet in Mice

The toxicity of* CpMet *was assessed in mice according to Lorke's model [24] with modifications. The animals were separated in six different groups (*n* = 10): one control group (vehicle; isotonic saline solution) and five different treatment groups. For the treatment groups,* CpMet* was orally administered during 20 consecutive days under one of the two following schemes: (1)* CpMet* (10, 100 or 1,000 mg/kg) administration once a day and (2)* CpMet* (100 or 300 mg/kg) administration twice a day. Five animals from each group were sacrificed immediately after the last vehicle or* CpMet* administration, and another five animals were euthanized 15 days after the last day of administration. Changes in body weight, behavior, and excretions (urinary, fecal, and mucus) were daily recorded and compared with negative controls. Blood samples were obtained by cardiac puncture for the determination of hematological (total erythrocyte count, hemoglobin, hematocrit, total and differential leukocyte count, platelet count, and total protein) and biochemical parameters (glucose, cholesterol, triglycerides, alanine amino transferase, aspartate amino transferase, alkaline phosphatase, bilirubin, urea, creatinine, BUN, and uric acid). Moreover, the mice were dissected and different organs (liver, kidneys, spleen, small bowel, stomach, and large bowel) were excised. Changes in weight and in the macroscopic morphology were recorded. For histopathology examination, the specimens were fixed and processed following standard histological techniques.

### 2.8. Gastric Ulcer Resolution

The effect of* CpMet* on the gastric ulcer resolution was evaluated as follows: the gastric ulcer was induced by intragastric instillation of ETOH (7 ml/kg). After 24 hours of recovery, mice were separated into 4 groups (*n* = 20 per group) and were orally treated for 20 consecutive days under one of the following experimental conditions: (1) VEH (isotonic saline solution, 7 ml/kg); (2) carbenoxolone (50 mg/kg), 2 doses per day; (3)* CpMet* (100 mg/kg), 2 doses per day; and (4)* CpMet* (300 mg/kg), 2 doses per day. Five mice from each group were sacrificed on the 5th, 10th, 15th, and 20th day after the beginning of the treatment and the gastric ulcer resolution was assessed by macroscopical, histopathological, and histochemical analysis of the stomachs. As it was done for the 20-day toxicity test, changes in body weight, behavior, and excretions (urinary, fecal, and mucus) were daily recorded and compared with negative controls.

The* CpMet* doses employed for the test were selected based on previous experiments in order to ensure the maximum effect on the resolution of gastric ulcers.

### 2.9. Histological Analysis

For the ethanol-induced gastric ulcer assessment, the stomachs were dissected out, insufflated with 2 ml of 10% neutral buffered formalin, and fixed in the same solution until the histological processing. The stomachs were opened throughout the great curvature. The glandular portion of the stomach was embedded in paraffin wax and sagittal sections of 5 *μ*m of thickness were obtained. Tissue sections were stained with hematoxylin-eosin (H&E) and periodic acid Schiff (PAS). For the toxicity histopathological examination, the specimens were fixed in 10% neutral buffered formalin and processed following standard histological techniques and stained with H&E. Tissue sections were examined under light microscope for morphological or cellular changes.

### 2.10. Statistical Analysis

Data are presented as mean ± SEM of *n* ≥ 6 per group and at least two independent replicates. Statistically significant differences between the treatments were tested by one-way ANOVA followed by Tukey's test. *P* < 0.05 was considered statistically significant. GraphPad Prism version 5.00 for Windows, GraphPad Software, La Jolla, California, USA (https://www.graphpad.com), was used for statistics and plotting.

## 3. Results

### 3.1. Gastroprotective Mechanism of CpMet

**Figure 1:**
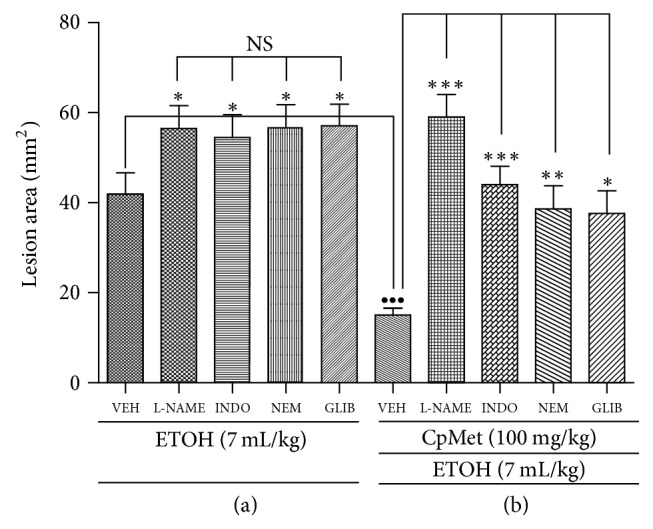
*CpMet gastroprotective mechanism in an ethanol-induced gastric ulcer model in mice.* (a) Protocol validation and (b) effect of* CpMet*.* CpMet*,* C. procera* methanolic extract 100 mg/kg; VEH, isotonic saline solution; L-NAME, N^*ω*^-nitro-L-arginine methyl ester 10 mg/kg; INDO, indomethacin 10 mg/kg; NEM, N-ethylmaleimide 1 mg/kg; GLIB, glibenclamide 5 mg/kg. Each column represents the mean ± SEM of three independent replicates (*n* = 6 per group). An ANOVA was performed followed by Tukey's test. For (a), ^**∗**^*P* < 0.05, significant difference versus control (VEH); NS, no significant difference between the four pretreatments. For (b), ^•••^*P* < 0.001, significant difference (VEH +* CpMet *+ ETOH) versus control (VEH + VEH + ETOH); ^**∗**^*P* < 0.05, ^**∗∗**^*P* < 0.01, ^**∗∗∗**^*P* < 0.001 significant difference between (pretreatment +* CpMet *+ ETOH) versus control (VEH +* CpMet *+ ETOH).

To determine the* CpMet* gastroprotective mechanism, a three-step murine protocol was performed: (1) pretreatment administration (with L-NAME, or INDO, or NEM, or GLIB), (2)* CpMet* administration (100 mg/kg), and (3) ETOH administration to induce gastric damage. In order to validate the protocol, each pretreatment was administered in a concentration intended to produce a significant higher damage than ETOH alone. As it can be seen in Figure 1(a), the lesion area induced by the four pretreatments did not show any statistical significant difference among them, but they were higher compared to control.

Figure 1(b) shows the results obtained when* CpMet* was administered after each pretreatment. Comparing the lesion areas induced by ETOH in the absence or presence of* CpMet* (VEH + VEH + ETOH versus VEH +* CpMet *+ ETOH), it can be observed that the extract reduced by 64.0 ± 1.56% the lesion area. Moreover, the four pretreatments with either L-NAME, INDO, NEM, or GLIB (Figure 3(b)) significantly attenuated or abolished the gastroprotective effect of* CpMet.*

### 3.2. Effect of CpMet on the pH of Gastric Contents of Ethanol Ulcerated Mice

**Figure 2:**
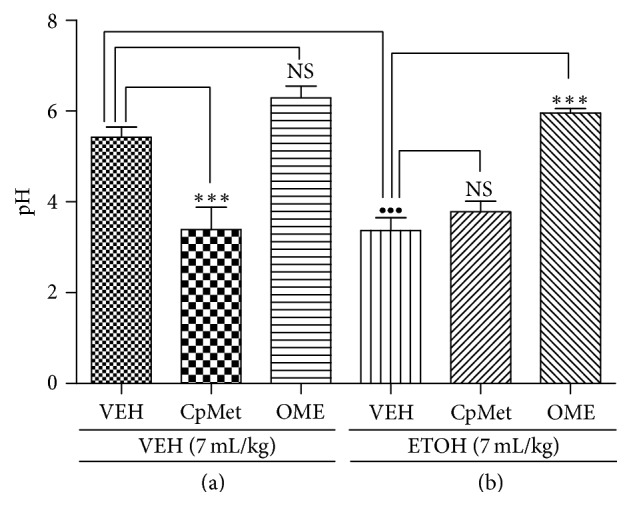
*Effect of CpMet on gastric pH value.* (a) Effect of* CpMet* and OME on basal gastric pH. (b) Effect of* CpMet* and OME on the pH value in ETOH-induced gastric injury. VEH, isotonic saline solution;* CpMet*,* C. procera* methanolic extract 100 mg/kg; OME, omeprazole 20 mg/kg. Each column represents the mean ± SEM of three independent replicates with 6 animals per group. An ANOVA was performed followed by Tukey's test. For (a), ^*∗∗ ***∗**^*P* < 0.001, significant difference versus control (VEH); NS, no significant difference versus control (VEH). For (b), ^•••^*P* < 0.001, significant difference of (VEH + ETOH) versus control (VEH + VEH); ^*∗∗ ***∗**^*P* < 0.001, significant difference versus control (VEH + ETOH); NS, no significant difference versus control (VEH + ETOH).

Figure 2(a) shows that the administration of* CpMet* by itself induced a significant pH reduction of the gastric contents (final pH = 3.6) compared to VEH (pH = 5.4) or omeprazole (pH = 6.1, no significant difference versus control group) treated animals. When the ETOH injury was imposed (Figure 2(b)), the pH values decreased significantly in the animals treated only with the vehicle.* CpMet* treated animals exhibited the same behavior as the vehicle. As expected, the treatment with omeprazole clearly reduced acid secretion under injurious conditions (Figure 2(b)).

### 3.3. CpMet Toxicity Evaluation

The lethality and the toxic adverse effects generated by prolonged* CpMet* oral administration were assessed considering the conditions of the gastric ulcer resolution assay (Section 3.4.).* CpMet* toxicity was evaluated in mice using the following treatment schemes during a 20-day continuous administration: (I)* CpMet* (10, 100, or 1,000 mg/kg) once a day and (II)* CpMet* (100 or 300 mg/kg) twice a day. Each group of animals was sacrificed in two stages: half of the group after the last administration (to analyze* CpMet* direct effects) and the other half, 15 days after the last treatment dose (to check animal recovery in the case that* CpMet* could produce any alteration).

Under the tested treatment schemes, no lethal effect was observed in any of the experimental animals. The hematological and biochemical parameters analyzed did not show significant alterations in the two sampling stages compared with the control groups (data not shown). Additionally, no significant abnormalities in body weight, behavior, macroscopic organ morphology (including weight), and histopathological appearance were detected (data not shown). Regarding body excretions, only changes in feces consistency (softness) were observed during the initial* CpMet* administration, but this condition was reversed after 5–7 days of continuous administration. In summary, a 20-day continuous* CpMet* regimen did not produce any clinical damage, supported by normal histopathological, hematological, hepatic, and renal function tests.

### 3.4. Effect of CpMet on the Resolution of Ethanol-Induced Gastric Ulcers

Once the toxicological safety of the extract is ascertained, its effect on the resolution of ethanol-induced gastric ulcers was evaluated. Mice were treated with* CpMet* for a 20-day continuous oral administration of 100 mg/kg (twice a day) or 300 mg/kg (twice a day). The effect of the extract on* GU* resolution was assessed on the 5th, 10th, 15th, and 20th day by macroscopical, histopathological, and histochemical analysis.

**Figure 3:**
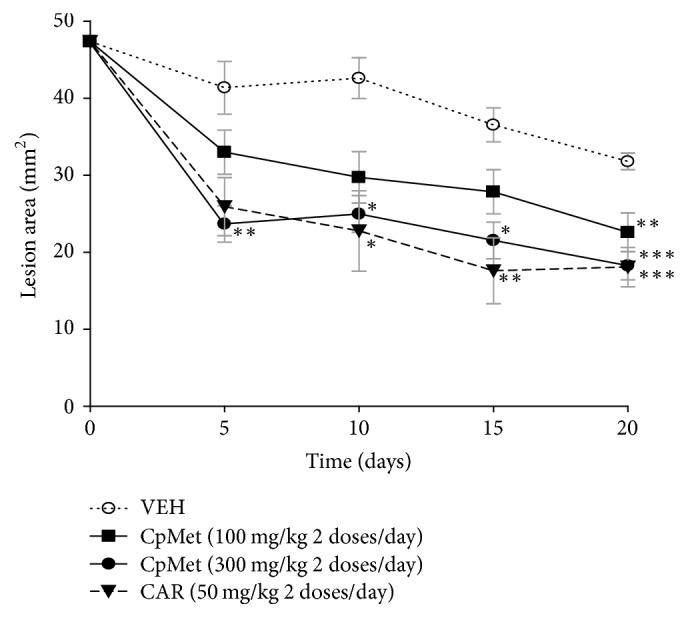
*Effect of CpMet on the resolution of ethanol-induced gastric ulcers in mice.* The graph shows the reduction of the lesion area over time, induced by a continuous 20-day administration of different treatments. VEH, isotonic saline solution;* CpMet*,* C. procera* methanolic extract; CAR, carbenoxolone. Each point represents the mean ± SEM of two independent replicates (*n* = 5 animals). An ANOVA analysis followed by Tukey's test was performed. ^**∗**^*P* < 0.05, ^**∗∗**^*P* < 0.01, ^*∗∗ ***∗**^*P* < 0.001, significant difference versus control (VEH at the corresponding time).

Figure 3 shows a reduction in the lesion area with each one of the treatments over time. As it can be seen, when VEH was administered to mice, there was a spontaneous ulcer resolution process, reaching on the 20th day a 35% diminution in the lesion area (from 48.89 ± 3.05 mm^2^ to 31.78 ± 0.78 mm^2^). Regarding the effect of* CpMet*, on the 20th day of the regimen, a 53.82% healing effect was observed with 100 mg/kg (twice a day) compared to the original damage (*t* = 0). When the dose was increased to 300 mg/kg, administered twice a day, the* CpMet* healing effect became more evident with respect to the previous dose used, affording a significantly 62.65% reduction in the area of the initial ulcer (from 48.89 ± 3.05 mm^2^ to 18.26 ± 1.45 mm^2^). The positive control CAR, at 50 mg/kg, twice a day, speeded up the healing of gastric ulcer in a similar way compared to the higher dose of* CpMet*, reducing the original wound area in a 63%.

Comparing the* CpMet* healing effect with the VEH, the 300 mg/kg dose, was the only treatment with statistical significance at all the sampled times (*P* < 0.05 to 0.001). The CAR treatment (positive control) was also significantly different compared to the VEH, except for the result recorded on the 5th day of sampling (Figure 3).

If we consider the remaining damage obtained at the 20th day with the VEH treatment (lesion area = 31.78 ± 0.78) as the 100%, we can calculate a net healing value of 42.54% with* CpMet* 300 mg/kg, twice a day. Performing the same calculation, a twice a day administration with 50 mg/kg of CAR, resulted in a 43.17% net healing.

#### 3.4.1. Macroscopic Examination of Stomachs

**Figure 4:**
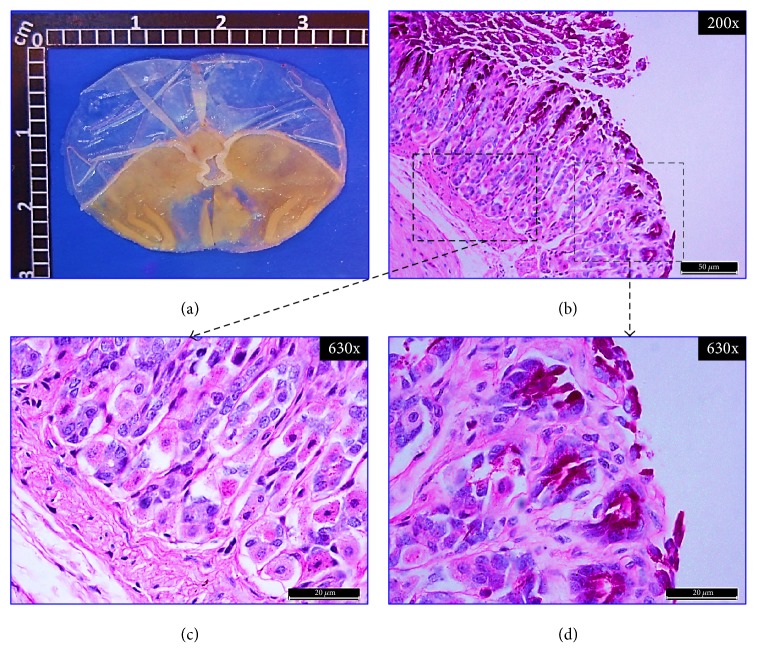
*Normal control.* Mice stomach appearance without any treatment. (a) Macroscopic view of a fresh dissected stomach without injuries. (b), (c), and (d) Bright-field microscopic views of 5 *μ*m thickness sagittal sections of the glandular portion of the stomach, stained with PAS. (b) Panoramic histochemical appearance showing tissue architectural integrity. Fuchsia clusters indicate PAS positivity and the presence of neutral mucus surrounding the mucosal epithelium. Dashed boxes: right, magnification to show mucus presence; left, histological status of the tissue. (c) Histological observation of the gastric mucosa showing well-organized mucosa structures without basal inflammatory infiltrate. (d) Histochemical analysis of the normal gastric mucosa of mice displaying PAS-positive neutral mucosubstances that predominate in the entire gastric surface.

**Figure 5:**
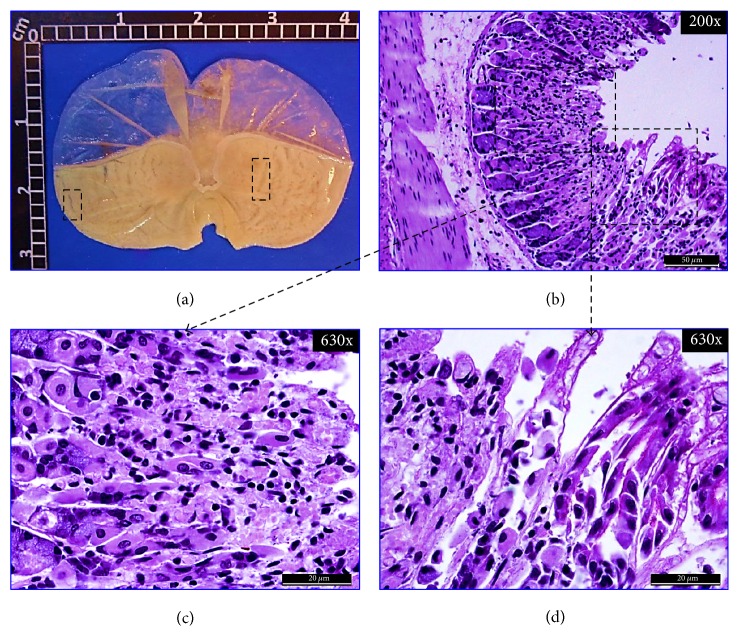
*Negative control.* Mice stomach treated with isotonic saline solution (7 ml/kg twice a day) on the 20th day after GU induction. (a) Macroscopic view of a formalin fixed stomach. Dashed boxes show the typical scars observed after 20 days of the GU induction with ethanol. (b), (c), and (d) Bright-field microscopic views of 5 *μ*m thickness sagittal sections of the glandular portion of the stomach, stained with PAS. (b) Panoramic histopathological appearance showing an extended degree of mucosal damage with disorganized architecture and basophilic staining due to persistent leukocyte infiltration. Dashed boxes: right, magnification to show mucus presence; left, histopathological status of the tissue. (c) Histopathological observation of the gastric mucosa revealing diffusive erosion of the gastric mucosa cell layer and abundant inflammatory cells present in the lamina propria. (d) Histochemical analysis presenting weak PAS positivity due to loss of epithelial continuity in the mucosa surface and gastric mucous neck cells.

**Figure 6:**
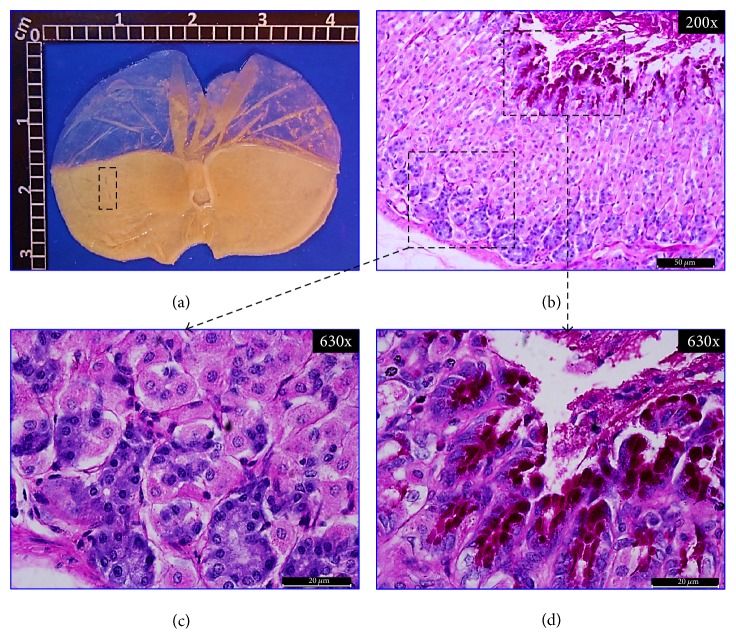
*Positive control.* Mice stomach treated with CAR (50 mg/kg, twice a day) on the 20th day after GU induction. (a) Macroscopic view of a formalin fixed stomach showing only some minor scars (dashed box). (b), (c), and (d) Bright-field microscopic views of 5 *μ*m thickness sagittal sections of the glandular portion of the stomach, stained with PAS. (b) Panoramic histopathological appearance showing robust basal architecture with some alterations at the apical portion of the mucosa and abundant clusters of PAS-positive staining regions. Dashed boxes: right, magnification to show mucus presence; left, histopathological status of the tissue. (c) Gastric mucosa revealing normal glandular organization without inflammatory infiltrate. (d) Gastric mucosa with strong PAS positivity surrounding the epithelia and areas of expanded surface mucous cells. In some areas, a slightly disrupted regeneration of the gastric epithelium is observed.

**Figure 7:**
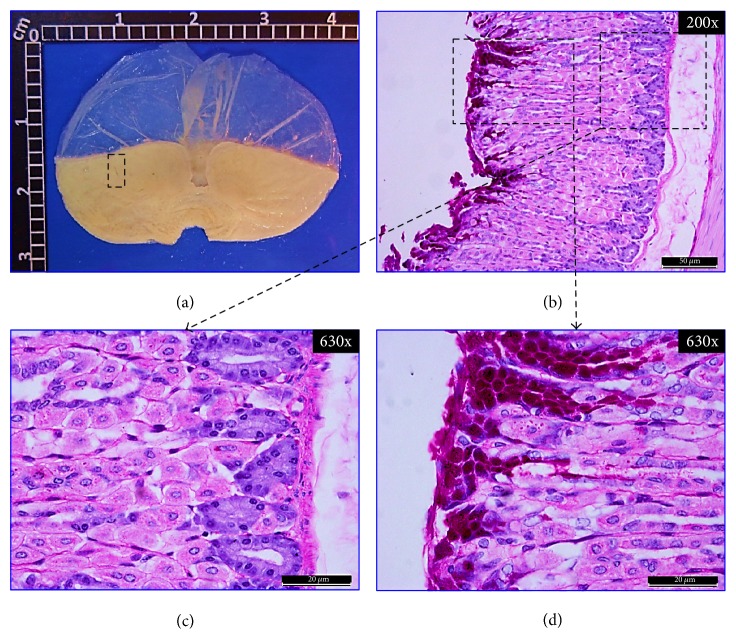
*CpMet treatment.* Mice stomach treated with* CpMet* (300 mg/kg twice a day) on the 20th day after GU induction. (a) Macroscopic view of a formalin fixed stomach mainly showing a smooth surface with few flat small scars (dashed box). (b), (c), and (d) Bright-field microscopic views of 5 *μ*m thickness sagittal sections of the glandular portion of the stomach, stained with PAS. (b) Panoramic histopathological appearance revealing a good organization of the basal gastric mucosa architecture with only minor alterations at the apical portion of the mucosa and abundant PAS-positive clusters lining the epithelium. Dashed boxes: left, magnification to show mucus presence; right, histopathological status of the tissue. (c) Histopathological observation displaying well-organized mucosal architecture, no inflammatory infiltrates are observed. (d) Gastric mucosa with strong PAS positivity surrounding the epithelium and areas of expanded mucous neck cells secreting increased amounts of mucins. Normal and well-defined mucous producing cells are observed.

Figure 4(a) shows a representative macroscopic image of a normal stomach with a smooth surface and no visible scars. In Figures 5(a), 6(a), and 7(a), representative stomach images, on the 20th day of the experiment, of controls and* CpMet* (300 mg/kg, twice a day) treated mice are depicted.  Figure 5(a) shows a stomach of the negative control group (VEH) where many ethanol-induced scars are visible. It has to be noticed that, after 20 days of the ulcer induction with ethanol, the lesions observed are usually characterized by the presence of rounded scars without the hemorrhage or edema of an acute ulcer.  Figure 6(a) presents a CAR (50 mg/kg, twice a day) positive control stomach, where a substantially improved appearance of the gastric surface compared to VEH (Figure 5(a)) is observed. The stomach is smoother and only a few small rounded scars are visible. Finally, in Figure 7(a), the stomach of the* CpMet* (300 mg/kg, twice a day) treated animals exhibits similar healing characteristics as the positive control (Figure 6(a)). This observation is supported by the statistical analysis where both treatments (CAR and* CpMet*) reached the same significance level (Figure 3).

#### 3.4.2. Histochemical and Histopathological Analysis

To ascertain the previous macroscopic observations, a histopathological analysis was performed. The histological inspection of a normal gastric mucosa exhibits the mucosal surface and the gastric pits lined by columnar epithelial cells as well as PAS-stained neutral mucins and no inflammatory cell infiltrate, indicating an intact gastric mucosa layer (Figures 4(b), 4(c), and 4(d)). Figures 5(b) and 5(d), corresponding to VEH treated mice on the 20th day of the experiment, show a significantly reduced PAS-positive adherent gastric mucus in the surface cells compared with normal mice (Figures 4(b) and 4(d)). Furthermore, diffusive erosion of the gastric mucosal cell layer with distorted and fragmented cells besides an increased amount of inflammatory cells throughout the mucosa and submucosa is observed (Figures 5(b) and 5(c)). In contrast, on the 20th day, the apical surface of the gastric mucous cells in CAR (50 mg/kg, twice a day) treated mice reveals a strong PAS-positive staining and a lack of inflammatory cells (Figures 6(b), 6(c), and 6(d)). On the other hand, compared with the control group (VEH),* CpMet* (300 mg/kg twice a day) treated mice exhibit an alleviation of the erosions in the gastric mucosal cell layer and an increased PAS reaction, indicating a higher production of mucosubstances (Figures 7(b) and 7(d)). In addition to the remarkable mucus production, no inflammatory (neutrophil/monocyte) infiltration of the mucosa is observed (Figures 7(b) and 7(c)). An almost similar pattern, without inflammatory infiltration and augmented mucus production evidenced by PAS-positive clusters lining the mucosal surface, is observed in the photomicrographs of* CpMet* 100 mg/kg, twice a day treated mice (data not shown). Nevertheless, the healing effect of this dose is not enough to alleviate the GU in a 20-day period as it could be perceived in the macroscopic images as well as by the presence of a slightly disrupted regeneration of the apical surface (data not shown).

## 4. Discussion

In a previous work, we reported the remarkable gastroprotective activity of* C. procera* bark methanolic extract* (CpMet)*, as well as its anti-*H. pylori* activity [21]. On this basis, the present study was performed to examine the mechanisms by which* CpMet* prevents gastric damage and to determine if a continued* CpMet* treatment promotes the resolution of preexisting gastric ulcers.

Considering that gastric ulcers develop as a result of an imbalance between defensive and injurious gastric mucosal factors, it was needed to assess the effect of* CpMet* on the gastric protective mechanisms.

There are several molecules that specifically block some of the endogenous gastroprotective mechanisms. Among the most studied and best characterized are L-NAME, INDO, NEM, and GLIB, involved with endogenous NO synthesis, prostaglandins (PGs) production, sulfhydryl groups, and ATP-sensitive potassium (K_ATP_) channels, respectively. As expected, the pretreatment with L-NAME, or INDO, or NEM, or GLIB effectively suppressed the targeted gastroprotective mechanism (Figure 1(a)). The administration of* CpMet* before the ethanol-induced injury clearly produced a remarkable gastroprotective activity (64.0 ± 1.56% reduction of the lesion area). Nevertheless, when the extract was administered after the four pretreatments, the antiulcer effect of* CpMet* was abolished or attenuated (Figure 1(b)), indicating that the extract gastroprotective activity is related to the four mechanisms evaluated. However, a multiple means comparison among the treatments showed a greater dependence on endogenous NO and PGs, followed by sulfhydryl groups and K_ATP_ channels.

Intragastric administration of absolute ETOH to experimental animals is a widely used model to induce gastric ulceration. The pathogenesis of ethanol-induced gastric ulcers is complex and provokes a broad spectrum of metabolic and functional changes, which include an increase in acid secretion, oxidative stress, and depletion of nonprotein sulfhydryl (NP-SH) compounds, diminishment of the gastric mucosal blood flow, impairment of the NO pathway, and decreased PGs synthesis [25–28].

NO has been recognized as an important mediator in the maintenance of gastric mucosal integrity [29], through the modulation of gastric mucosal blood flow, mucus, and bicarbonate secretions [30]. Thus, the impairment in NO synthesis affects the gastric microcirculation [25]. In the present work, the inhibition of NO synthesis with L-NAME clearly increased the lesion area induced by ETOH administration. In the group receiving* CpMet* with the NO-synthase inhibitor, the lesion area remained the same as in the untreated group (without the extract) (Figure 1). This data suggests that the maintenance of NO production is crucial for the gastroprotective mechanism of* CpMet*, perhaps by preventing gastric microcirculation damage.

PGs serve as gastric cytoprotectants by attenuating or preventing mucosal lesions. In fact, the inhibition of their synthesis by blocking the cyclooxygenase enzyme with NSAIDs such as indomethacin results in the reduction of gastric mucosal blood flow and mucosal damage. In the present study, the gastroprotection favored by* CpMet* was attenuated by INDO pretreatment. The same behavior was attained when blocking K_ATP_ channels with GLIB. The protective effect exerted by PGs is, at least in part, mediated by the activation of K_ATP_ channels [31], contributing to improved gastric microcirculation. Regarding that the lesion area generated in the presence of* CpMet* is sensitive to both INDO and GLIB, it is likely that the partial gastric protection afforded may be related to maintaining a moderate production of PGs, which in turn, act as K_ATP_ channel activators.

NP-SH compounds protect gastric mucosa by controlling mucus production and binding free radicals. Previous reports have shown that NP-SH compounds are depleted in ETOH-induced gastric lesions [32]. In this work, the ulcerogenic effect mediated by the administration of NEM and ETOH was partially reduced by* CpMet* treatment, suggesting that, in the gastroprotective mechanism of the extract, probably underlies the elimination of the noxious free radicals through the participation of endogenous NP-SH.

Regarding the effect of* CpMet* on gastric pH (Figure 2), our results suggest that the extract did not have a favorable effect on this parameter; however it is noteworthy to mention that the administration of the extract did not intensify the acidity of gastric contents induced by ETOH alone, probably due to a local effect of* CpMet* that prevents ETOH absorption and its outcomes on gastric pH. The ulcerogenic effect of ETOH is based on an increase in the secretion of histamine, pepsin, and H^+^ ions, among other deleterious elements; nevertheless, ethanol-induced gastric ulcers are not inhibited by antisecretory drugs such as cimetidine [33]. In this sense, it appears to be more important to improve the mucosal defensive mechanisms rather than just blocking the acid production. On the basis of our data, it can be suggested that the gastroprotective effect of* CpMet* does not rely on the gastric acid inhibition; however, it cannot be ruled out its antiulcer potential even in the absence of an acid antisecretory effect, since the extract could serve as a coadjuvant of conventional antiacid treatments. Moreover, the absence of an antiacid mechanism of* CpMet* encourages its beneficial protective effects, taking into account the fact that long-term therapies based on modifications of acid secretion are related to adverse side effects (e.g., hypergastrinemia) due to augmented pH in the gastric lumen [33].

Ethanol administration induces gastric ulcers that spread over large areas: lesions that are characterized by persistent leakage and increased blood flow stasis at the ulcer margin accompanied by edema, congestion of the surface epithelium, and inflammatory infiltration. The assessment of* CpMet* effect on the resolution of gastric ulcers induced by ethanol revealed that a 20-day continuous oral administration of the extract at dose of 300 mg/kg, twice a day, clearly reduced the lesion area (62.65%) (Figure 3); it has to be pointed out that the main effect relied on the time required to significantly reduce the initial lesion area, which was only about 5 days. Thus, comparing the ulcer resolution obtained on the 5th day with* CpMet *300 mg/kg (twice a day) and the negative control (VEH), a 42.73% of improvement was attained, while 20.23% of healing was reached with 100 mg/kg, 2 doses per day. This data shows a dose-dependent effect and indicates the necessity of a high and reinforced* CpMet* dose (300 mg/kg, twice a day) to get a significant early gastric resolution. It has to be emphasized that this latter dose of* CpMet* administered twice a day is the one that ensured the maximum healing effect, since previous dose-response experiments (data not shown) revealed that single doses per day of the extract were not enough to obtain the resolution effect. Further studies are required to elucidate the pharmacokinetics and pharmacodynamics of* CpMet*, which may explain the necessity of a twice daily dose, besides being a critical requirement for developing a safe and effective phytomedicine.

Numerous studies have shown that current antiulcer drugs, targeted to inhibit acid secretion, are insufficient to promote a complete ulcer healing and to prevent the relapse of gastric ulcers. The resolution of a gastric ulcer involves the formation of a scar through migration and proliferation of epithelial and connective tissue cells, besides active angiogenesis and extracellular matrix deposition [13]. It has been suggested that the quality with which this repair process occurs plays a key role in the risk of ulcer recurrence. On this basis, the concept of quality of ulcer healing (QOUH) based on the histological maturity of regenerated mucosa of healed ulcers has been proposed. PGs and some growth factors (e.g., VEGF) have been positively associated with an improved QOUH, whereas the persistence of an increased infiltration with neutrophils and macrophages results in an immature regenerated area with distorted architecture and prone to ulcer recurrence [11].

When an external aggression, such as ethanol, alters the gastric mucosa homeostasis, gross histological changes linked to the ulcerative process can be observed. Although, gastric mucosa activates different responses to alleviate damage, after 20 days of GU induction, the mucosal recovery is not fully completed. In the context of the present work, the results show that* CpMet *(300 mg/kg, twice a day) has a strong cicatrisation activity by reaching a net healing value of 42.54% (on the 20th day), a percentage that is even afforded since the 5th day of treatment (Figure 3). Moreover, unlike VEH treatment, the stomachs of* CpMet* (300 mg/kg, twice a day) treated mice exhibited flat scars with an increased mucus production and most importantly, without inflammatory infiltration, which seems to be the basis of a good QOUH. In essence, the flat macroscopical appearance of the scars generated with* CpMet* treatment dovetails with the histochemical findings, by showing a marked improvement in the gastric ulcer resolution. Further research should be done to clarify the precise mechanism by which the extract exerts its effect on the resolution of gastric ulcers; however, in the light of our results, it seems that* CpMet* has a potent anti-inflammatory effect by limiting the attraction and accumulation of inflammatory cells.

There are many works that have sought the cure of gastric ulcers by testing compounds derived from plants; however, herbal extracts have shown to be a better treatment option, or an excellent choice as adjuvants, to prevent or promote an effective healing effect due to the multiple activities they can exert simultaneously (i.e., antibiotic, antiulcerogenic, anti-inflammatory, antioxidant, angiogenic, and cytoprotective) [12, 13]. Referring specifically to extracts or preparations from plants, there are several studies that have assessed their gastroprotective and ulcer healing activities. Indeed, many of these works have demonstrated that the efficacy to prevent or ameliorate gastric ulcers is comparable or even better than that of some drugs used in conventional therapies (i.e., sucralfate, omeprazole, or cimetidine). However, the beneficial effects vary extensively depending on the injurious agent used to induce the ulceration (NSAID's, ethanol, acetic acid, cold water restraint stress, HCl, or pyloric ligation), the plant species, type of extract, and the treatment duration. For instance, in a review performed by Bi et al. [12], the percentage of efficacy in treating gastric ulcers with only one or a combination of herbal extracts ranged from 20 to ~80%. In another study performed by Mota da Silva et al. [34], an hydroalcoholic extract of* Maytenus robusta* reduced the gastric ulcer area by 53%, and the healing effectiveness seemed to be mediated by increased gastric mucin content and reduced oxidative stress and inflammatory parameters at the site of the ulcer; nevertheless, it seems that the extract did not reduce the leukocyte migration as in the case of* CpMet*.

Although it is true that there are a vast number of studies in the literature reporting that diverse herbal extracts promote a significant reduction of gastric ulcers, some of the results are mainly based on the macroscopic measurement of the ulcerated area, leaving aside the histological assessment, which is the cornerstone to ascertaining a good QOUH. Furthermore, it is noteworthy that only a few herbal extracts have available information related to their toxicological safety, which is essential to promote the safe use of herbal medicines.

According to the data obtained in the present study and our previous report [21],* CpMet* exerts polypharmacological activities, demonstrated by the following findings: (1)* in vitro* anti-*H. pylori* activity; (2) gastroprotective and ulcer healing effects; and (3) anti-inflammatory action. Additionally, the extract turned out to be safe under subacute administration which, besides its beneficial properties, makes it an attractive candidate to continue its study in preclinical tests. The advantage that* CpMet *has polypharmacological effects is that it can impact on various known etiological factors of gastric ulcers as well as in the mechanisms that underlie the ulcer resolution. The* CpMet* anti-*H. pylori* activity is of great importance considering that this bacterium is responsible for 70–85% of gastric ulcers [35]. Moreover, the benefits of the extract are related, in a greater or lesser extent, to NO synthesis, PGs production, sulfhydryl groups, and K_ATP_ channels, mechanisms that, in general terms, modulate the gastric defenses such as microcirculation, mucus, and ROS production. The processes that mediate the protection of the gastric mucosa are neither independent nor different from the ones that participate in the resolution of gastric ulcers. In this sense, the antiulcer and healing effects of* CpMet* may rely on an increased mucus production, an anti-inflammatory action, and a partial dependence on PGs, compounds that promote a high QOUH.

## 5. Conclusion

The data obtained in the present work highlights the effectiveness of the methanolic extract of* C. procera* for improving the quality of resolution of a preexisting ulcer by different mechanisms, but mostly through cytoprotective and anti-inflammatory effects. Even though conventional treatments are effective in the management of gastric ulceration, they are not sufficient to avoid ulcer recurrences. Thus,* CpMet* could be a good adjuvant of current therapies, based on its antimicrobial, antiulcer, and gastric healing effects.

## Abbreviations

CAR: Carbenoxolone
CpMet: * Cyrtocarpa procera* methanolic extract
ETOH: Absolute ethanol
GLIB: Glibenclamide
GU: Gastric ulcers
H&E: Hematoxylin-eosin
INDO: Indomethacin
K_ATP_: ATP-sensitive potassium
L-NAME: N^*ω*^-Nitro-L-arginine methyl ester
NEM: N-Ethylmaleimide
NO: Nitric oxide
NP-SH: Nonprotein sulfhydryl
NSAIDs: Nonsteroidal anti-inflammatory drugs
OME: Omeprazole
PAS: Periodic acid Schiff
PGs: Prostaglandins
QOUH: Quality of ulcer healing
ROS: Reactive oxygen species
VEH: Vehicle.
